# Mass community cycling events: Who participates and is their behaviour influenced by participation?

**DOI:** 10.1186/1479-5868-3-39

**Published:** 2006-11-07

**Authors:** Heather R Bowles, Chris Rissel, Adrian Bauman

**Affiliations:** 1 Centre for Physical Activity and Health, University of Sydney Medical Foundation Building (K25) Level 2, 94 Parramatta Road, Camperdown NSW 2050, Australia; 2 Health Promotion Service, Sydney South West Area Health Service and School of Public Health, University of Sydney. Level 9, King George V, Missenden Road, Camperdown NSW 2050, Australia

## Abstract

**Background:**

Participation in mass physical activity events may be a novel approach for encouraging inactive or low active adults to trial an active behaviour. The public health applicability of this strategy has not been investigated thoroughly. The purpose of this study to was describe participants in a mass cycling event and examine the subsequent effect on cycling behaviour.

**Methods:**

A sample of men and women aged 16 years and older (n = 918) who registered online for a mass cycling event reported cycling ability and number of times they rode a bicycle during the month before the event. One month after the event participants completed an online follow-up questionnaire and reported cycling ability, lifestyle physical activity, and number of times they rode a bicycle during the month after the event. McNemar's test was used to examine changes in self-rated cycling ability, and repeated measures mixed linear modeling was used to determine whether average number of monthly bicycle rides changed between pre-event and post-event assessment.

**Results:**

Participants in the cycling event were predominantly male (72%), 83% rated themselves as competent or regular cyclists, and 68% rated themselves as more active than others of the same sex and age. Half of the survey respondents that rated their cycling ability as low before the event subsequently rated themselves as high one month after the event. Respondents with low pre-event self-rated cycling ability reported an average 4 sessions of bicycle riding the month before the event and an average 6.8 sessions of bicycle riding a month after the event. This increase in average sessions of bicycle riding was significant (p < .0001). Similarly, first-time participants in this particular cycling event significantly increased average sessions of cycling from 7.2 pre-event to 8.9 sessions one month after the event.

**Conclusion:**

Participants who were novice riders or first time participants significantly increased their number of bicycle rides in the month after the event. Further knowledge about the public health applicability of mass events is needed, and methods for attracting less active and novice individuals to participate remain to be developed.

## Background

Almost half the population of Australia is insufficiently active to achieve recommended levels of physical activity [1]. Strategies that encourage sustained increases of physical activity at the population level are needed, and this applies to most western countries. Increasing population levels of cycling, either for recreation or transport, has considerable potential to increase physical activity. In Australia, cycling is the fourth most popular physical activity after walking, aerobics and swimming [2], with annual participation in cycling growing by 15.3% from 2001 to 2004. Almost half of Australian households have access to a bicycle, with bicycle ownership varying from 39% in Sydney to 65% in Canberra [3].

Strategies that encourage cycling range from individual or group education skills programs, mass media education programs, infrastructure programs that separate people riding bicycles from motorists, and traffic calming approaches which seek to slow the speed of motor traffic [4]. Community events, such as organized mass bicycle rides, are another approach that combines a large group participation program with mass media, as well as temporary infrastructure modifications and traffic calming through police support.

There is substantial interest in the potential for elite sporting events with mass exposure, such as the Olympic Games, to positively influence community interest in physical activity [5]. However, there is very little evidence that elite sporting events contribute to general population participation in physical activity [5], and the 2000 Sydney Olympics showed no effect on physical activity prevalence [6]. In fact, just watching elite sport may be hazardous to health [7].

Less is known about the role of participation in mass community events that are sub-elite. Large-scale fun runs, marathons, walking and cycling events are carried out in many cities, often with sponsorship and fundraising objectives. The distances are accessible, and these events are promoted to all segments of the general community [8]. The unanswered research question is whether these events have public health potential for influencing some sedentary individuals to trial physical activity, or whether they mostly attract those who are already regular runners and cyclists. Further, does participation in such community events lead to engagement with the activity, and short term maintenance of the specific physical activity behaviour post event? The purpose of this study was to examine the attributes of the participants and the effect of participation in a mass community cycling event on cycling behaviour.

## Methods

### Study Design

A subsample of online registrants in a mass cycling event in Sydney, Australia, completed a pre-event and post-event survey about cycling and physical activity. The cycling event is an annual scenic ride across the heart of Sydney organized by the non-government cycling advocacy organization, Bicycle New South Wales. Participants have the option to cycle 20 kilometers or 50 kilometers. The pre-event survey was part of the online event registration form and assessed entrants' self-rated cycling ability and physical activity level. The pre-event survey also included a question seeking permission for follow-up after the event. Informed consent was obtained by registrants' willingness to be contacted again for follow-up. Respondents were approached one month after the mass cycling event to complete the post-event survey. The post-event survey was administered via the Internet. Self-rated cycling ability and physical activity level were reassessed, as well as lifestyle physical activity in the last week. Participants received an email with a brief explanation of the survey, and a link to the survey for completion. Survey completion was voluntary, participants did not receive compensation for responding, and they could end the survey at any time. The study procedures were approved by the executive board of Bicycle New South Wales.

### Study Participants

Men and women aged 16 and older were selected for post-event survey from the pool of online registrants that consented to follow-up. Approximately 8,000 men and women participated in the cycling event, and 5,058 registered for the event online. The majority of online registrants were experienced cyclists (83%). The online registrants were significantly (p < .05) younger (32% aged 16–34, 64% aged 35–64, 4% aged 65 or older) than registrants who entered the event via mail-back form (23% aged 16–34, 72% aged 35–64, 5% aged 65 or older). The proportion of women was also significantly higher (p < .05) among online registrants (28%) compared to mail-back registrants (23%). Mail-back registrants did not provide any description of their cycling ability. Unfortunately, registrants via mail-back form were not asked for permission to be contacted for follow-up after the event, and were not included in this study.

Examining change in cycling behaviour among sedentary or low ability participants was the main objective of this study; however, the proportion of participants who self-rated their physical activity or cycling ability as low on the pre-event survey was small (13%). Therefore, to obtain the maximum number of responses from the subgroup of interest, participants were automatically selected for follow-up if they indicated they had low cycling ability or low physical activity level on the pre-event survey. Participants were also automatically selected for follow up if they were female to maximize the number of responses from women because women were underrepresented in the total sample (28%). A random sample of men and experienced cyclists were selected to comprise the remainder of the follow-up cohort. Of 3,325 online registrants who consented to follow-up, 2,068 were sampled to complete the post-event survey, and 1,135 (54.9%) responded.

There was no difference in the proportion of women, persons aged 65 or older, or persons with low physical activity level between responders to the follow-up survey and non-responders. Non-responders were more likely (p < .05) to be aged 16–34 (35%) than responders (25%), and non-responders were less likely to be experienced cyclists (76%) compared to responders (85%); however, there was no difference between responders and non-responders who were not experienced cyclists in the number of times they rode their bicycle the month before the event.

Respondents with missing data pertaining to mass cycling event participation history (n = 58), post-event self-rated cycling ability and/or physical activity level (n = 126), post-event number of monthly bicycle rides (n = 3), and post-event lifestyle physical activity (n = 30) were excluded from analysis, resulting in a final analytic sample of 918 respondents.

### Self-Reported Cycling Ability and Physical Activity

When participants registered online prior to the October 2005 mass cycling event they were asked, "How would you describe yourself as a cyclist?" Response choices were novice or beginner, occasional but tentative rider, occasional but competent rider, or regular rider. Participant self-rated cycling ability was dichotomized as either low (novice or tentative rider) or high (competent or regular rider). Participants described their physical activity level compared to others of the same sex and age as much more physically active, more physically active, about the same, less physically active, or much less physically active. Self-rated physical activity level was also dichotomized as low (much less or less active) or high (the same, more, or much more active). Finally, participants reported the number of times they rode their bicycle in the past month and if they had ever participated in this mass cycling event before.

One month after the mass cycling event occurred, the post-event survey was administered. Post-event survey respondents were asked the same questions regarding cycling ability and physical activity level as on the pre-event registration form. Post-event self-rated cycling ability and physical activity level were dichotomized as low or high by the same cut points as for the pre-event assessment. Participants were also asked to report the number of times they rode their bicycle during the month after the mass cycling event.

In addition to post-event cycling ability and self-rated physical activity level, questions from the Active Australia, a national survey of physical activity, were used to assess the amount of lifestyle physical activity respondents engaged in the week prior to the post-event survey [6]. Respondents reported the number of sessions and the amount of total weekly time they spent in walking, moderate-, and vigorous-intensity physical activities.

Physical activity assessed by the Active Australia was categorized as "sufficiently active" or "insufficiently active" in accordance with guidelines outlined by the Australian Institute of Health and Welfare [9]. Minutes per week spent in vigorous-intensity physical activity was weighted by a factor of two to account for additional benefits derived from higher intensity activity, then summed with minutes per week spent in walking and moderate-intensity activity to derive minutes per week of total activity. Respondents were categorized as "sufficiently active" if they participated in 150 minutes of total activity over five or more sessions in the previous week. If they did not meet these criteria they were categorized as "insufficiently active".

### Statistical Analysis

General descriptive and multivariate statistical analyses were performed using SAS (Version 9.1, 2000, SAS Institute Inc., Cary, NC). Data are presented as medians with interquartile range or means with standard error for continuous variables and counts (percentages) for categorical variables. McNemar's test was used to examine changes in self-rated cycling ability before the mass cycling event and after. Repeated measures mixed linear modeling (a generalization of standard linear modeling) was used to determine whether average number of monthly bicycle rides changed between pre-event and post-event assessment among participants with low baseline cycling ability and among first-time participants in the mass cycling event. Respondent sex, age, and (for first time participants) pre-event level of cycling ability were included in the models and tested as confounders. Non-significant covariates were removed from the final models. The significance level was set at p < .05.

## Results

The total pool of online registrants were more likely to be male (72%) than the subsample of respondents with complete post-event survey data (66%), as displayed in Table 1. Respondents to the post-event survey were also slightly more middle-aged, and of higher baseline self-rated cycling ability. The prevalence of low self-rated physical activity level was the same between the total sample and the subsample (3.7%). Nearly half of the post-event survey respondents were first-time participants in the mass cycling event. Most of the participants were competent or regular cyclists (83% of all online registrants, 85% of post-event survey respondents).

**Table 1:** Pre-event characteristics of mass cycling event online registrants and registrants who completed a post-event survey

	All Online Registrants (N = 5058)		Online Registrants Who Completed Post-Event Survey (N = 918)	
		
Baseline Characteristic	n*	%	n	%
				
Sex				
Male	3654	72.2	603	65.7
Female	1404	27.8	315	34.3
Age Group				
16–34 years	1630	32.3	231	25.2
35–49 years	2120	41.9	349	38.0
50–64 years	1107	21.9	307	33.4
65+ years	197	3.9	31	3.4
Self-Rated Cycling Ability				
Novice or Beginner	177	4.8	30	3.3
Occasional but Tentative	448	12.3	105	11.4
Occasional but Competent	1446	39.6	323	35.2
Regular Cyclist	1581	43.3	460	50.1
Self-Rated Level of Physical Activity				
Much Less Active	13	0.4	3	0.3
Less Active	110	3.3	31	3.4
About the Same	956	28.6	214	23.3
More Active	1614	48.4	468	51.0
Much More Active	645	19.3	202	22.0
Participated in this Cycling Event Before				
No	1715	49.2	399	43.5
Yes	1771	50.8	519	56.5

*Category n may not sum to N = 5058 because of missing data on entry forms.

Presented in Table 2 are the frequencies of low and high post-event self-rated cycling ability by pre-event level. One month after the mass cycling event, 51.1% of respondents who were low pre-event self-rated cycling ability subsequently improved to a high self-rated level. Nearly 4% of those with a high pre-event level regressed to a low post-event level of self-rated cycling ability. McNemar's test indicated the probability of shifting from low level to high level was significantly higher than shifting from high level to low level (p < .0001). Although the number of respondents with low pre-event self-rated physical activity level was small (n = 34), similar patterns were observed (data not shown). Sixty-two percent of low active respondents pre-event improved to a high self-rated physical activity level, but 2% of highly active respondents regressed to a low post-event level.

**Table 2:** Self-rated level of cycling ability post-event, stratified by pre-event level

Pre-event: LOW (N = 135)				Pre-event: HIGH (N = 783)			
	
Post-event: Low		Post-event: High		Post-event: Low		Post-event: High	
			
n	%	n	%	n	%	n	%
			
66	48.9	69	51.1	29	3.7	754	96.3

Levels of lifestyle physical activity by self-rated cycling ability are presented in Table 3. Self-rated cycling ability were ordered as low pre-event/low post-event, high pre-event/low post-event, low pre-event/high post-event, and high pre-event/high-post event. The prevalence of sufficient lifestyle activity increased with self-rated cycling ability. Respondents with low self-rated cycling ability at both baseline and follow-up had the lowest prevalence of sufficient activity (75.8%), and respondents who maintained a high level at both baseline and follow-up had the highest prevalence (85.7%).

**Table 3:** Lifestyle physical activity one month post-event by self-rated level of cycling ability

		Pre-Event/Post-Event Self-rated level of cycling ability			
		
		Low/Low (n = 66)	High/Low (n = 29)	Low/High (n = 69)	High/High (n = 754)
					
		%	%	%	%
				
Insufficiently Active	142	24.2	27.6	14.5	14.3
Sufficiently Active	776	75.8	72.4	85.5	85.7

Among respondents with low pre-event self-rated cycling ability, the average number of monthly bicycle rides increased from four in the month before the event to seven in the month after the event (p < .0001), adjusted for sex of the respondents and shown in Table 4. Age was not a significant predictor and was removed from the model. Among first-time participants in the mass cycling event the average number of monthly bicycles rides also increased significantly (p < .0001), after adjusting for pre-event level of cycling ability. During the month prior to the event, first-time participants rode their bicycle an average 7.15 times, and rode an average 8.89 times during the month after the event. Neither respondent sex nor age were significant predictors and were removed from the final model. Similar results were observed for respondents with low pre-event self-rated physical activity level (data not shown), with an increase from an average 5 sessions the month before to an average 8 sessions the month after the event (p < .0001).

**Table 4:** Changes in the mean number of times cycled in the months pre- and post-event

		Pre-Event # rides/mo	Post-Event # rides/mo			
						
Group	n	Mean (SE)	Mean (SE)	Difference	t (p)	df
						
Low pre-event cycling ability	135	4.00 (0.40)	6.82 (0.62)	2.82	5.25 (<.0001)	133
First-time participant in this cycling event	399	7.15 (0.43)	8.89 (0.48)	1.73	5.57 (<.0001)	397

## Discussion

The results indicate that participants who were novice riders or were first time participants in this particular cycling event significantly increased their number of bicycle rides in the month after the event. Half of the participants who rated their level of cycling ability low at baseline then rated themselves as high a month after the mass cycling event.

The participants in a Sydney mass cycling event were more likely to be male, middle aged, cycling-experienced, and most were considered sufficiently physically active for a health benefit by population health standards. Approximately 85% of respondents to the follow-up questionnaire reported engaging in 150 minutes of physical activity over five or more sessions, compared to the 2005 state estimate of 52% of all New South Wales residents aged 16 years and older (57% of males, 47% of females) [10]. However, the finding that the prevalence of sufficient physical activity was much higher among respondents with low pre-event/high post-event cycling ability than among respondents with low pre-event/low post-event cycling ability warrants future investigation. The event targeted cycling activity, but may have contributed to the difference in lifestyle physical activity observed between these groups.

A small proportion of event participants rated their level of cycling ability as high at baseline, but subsequently rated themselves as low a month after the event. This could reflect injury or acute illness rather than true behavioural relapse. Studies of endurance runners have found an increased risk of illness during periods of heavy training and in the weeks following participation in a mass event [11,12]. Information about the epidemiology of injury and illness among participants in mass bicycling events is scant and comes from studies of events with distances longer than the Sydney mass cycling event. Serious injuries associated with participation in a 42-mile (67.59 kilometer) mass cycling event were uncommon, with only 5 injuries per 1,000 bicyclists, 95% of which were bruises and abrasions [13]. Data from long, multi-day bicycling tours indicate most injuries associated with participation are overuse injuries [14,15]. The repetitive mechanics of bicycling make cyclists susceptible to lower extremity injuries, the most common types being injuries to the knee, usually attributable to riding with excessive force [16]. To lower the risk of illness or overuse injury, a heavy training load can be accomplished with fewer negative health outcomes if training programs are designed to minimize strain and monotony [17]. Training materials were provided to participants in the Sydney mass cycling event through the event website.

There are limitations to the application of these results, including the small number of low active participants, which prevented a comprehensive analysis to examine change in self-rated physical activity in the low active subsample. Reliance on self-reported physical activity data is another limitation. However, the self-rated physical activity question used in this study has been validated previously [18], and we compared self-rated physical activity level with total minutes of physical activity assessed by the Active Australia questions, and found significant differences (p < .05) in mean number of weekly minutes of physical activity across the self-rated categories. The lower response rate (55%) of the follow-up internet survey raises the possibility that the respondents were the most positive ride participants, and participants who did not enjoy their ride experience or may have decreased their cycling frequency chose not to respond to the follow-up survey. Lack of power may have contributed to the inability to detect confounders in the regression models. Despite these limitations, this study is one of the first to examine the public health potential of a mass cycling event to promote adoption and maintenance of physical activity in a community.

Few mass participation events, related to bicycling, running or any other sport, have ever been evaluated to determine the effects upon population levels of physical activity. [8]. Prior mass event research has focused on process evaluation indicators, such as participant numbers or finishing times. Community level events, such as the mass cycling event examined by this study, could be used as part of integrated physical activity promotions if they attract less active and novice individuals to participate. Most participants in the Sydney cycling event were competent or regular cyclists, suggesting an event organizational orientation towards elite or competitive participation over the public health potential for increasing community participation. However, mass events have the potential to encourage participants to trial new behaviours in a non-competitive, controlled, and enjoyable environment, as well as have a broader agenda setting role across the community.

Other kinds of mass participation events have demonstrated the potential to attract inactive adults or adults with little cycling experience, such as single-day health promotion events to encourage physically active commuting instead of vehicle use for transportation. For example, participation in the Victoria Ride to Work Day has more than doubled since 2002, and attracted over 5,000 participants in 2005, approximately a fifth of whom were first time riders [19]. Other structured health promotion events also seem to have the potential to increase walking for transportation [20].

Further knowledge about the public health applicability of mass events is needed, and methods for attracting less active and novice individuals to participate remain to be developed. Partnerships between public health agencies and event organizers could result in promotional activities to encourage less active community members to trial new physical activity behaviours. This would require shared planning and marketing, and clear targeting of the less regularly active. Shorter event options, post-event reinforcement (including post-event promotions and additional events) may also be required in order to maintain motivation for these new event participants. If these partnerships were explored they could provide a novel approach to reaching inactive adults at the population level, and encourage many of them to contemplate trialing an active behaviour.

## Competing interests

The author(s) declare that they have no competing interests.

## Authors' contributions

HRB participated in the design of the study, collected the survey data, performed the statistical analysis, and helped to draft the manuscript. CR participated in the design of the study and helped to draft the manuscript. AB conceived of the study, participated in its design, and helped to draft the manuscript. All authors read and approved the final manuscript.
